# Immune system deregulation in hypertensive patients chronically RAS suppressed developing albuminuria

**DOI:** 10.1038/s41598-017-09042-2

**Published:** 2017-08-21

**Authors:** Marta Martin-Lorenzo, Laura Gonzalez-Calero, Paula J. Martinez, Montserrat Baldan-Martin, Juan Antonio Lopez, Gema Ruiz-Hurtado, Fernando de la Cuesta, Julián Segura, Jesús Vazquez, Fernando Vivanco, Maria G. Barderas, Luis M. Ruilope, Gloria Alvarez-Llamas

**Affiliations:** 1 Departament of Immunology, IIS-Fundacion JimenezDiaz, REDinREN, Madrid, Spain; 2 Department of Vascular Physiopathology, Hospital Nacional de Paraplejicos SESCAM, Toledo, Spain; 3 Laboratory of Cardiovascular Proteomics CNIC, Madrid, Spain; 4 Hypertension Unit, Instituto de Investigación imas12, Hospital Universitario 12 de Octubre, Madrid, Spain; 5 Department of Biochemistry and Molecular Biology I, Universidad Complutense, Madrid, Spain; 6 Universidad Europea, Madrid, Spain

## Abstract

Albuminuria development in hypertensive patients is an indicator of higher cardiovascular (CV) risk and renal damage. Chronic renin-angiotensin system (RAS) suppression facilitates blood pressure control but it does not prevent from albuminuria development. We pursued the identification of protein indicators in urine behind albuminuria development in hypertensive patients under RAS suppression. Urine was collected from 100 patients classified in three groups according to albuminuria development: (a) patients with persistent normoalbuminuria; (b) patients developing de novo albuminuria; (c) patients with maintained albuminuria. Quantitative analysis was performed in a first discovery cohort by isobaric labeling methodology. Alterations of proteins of interest were confirmed by target mass spectrometry analysis in an independent cohort. A total of 2416 proteins and 1223 functional categories (coordinated protein responses) were identified. Immune response, adhesion of immune and blood cells, and phagocytosis were found significantly altered in patients with albuminuria compared to normoalbuminuric individuals. The complement system C3 increases, while Annexin A1, CD44, S100A8 and S100A9 proteins showed significant diminishment in their urinary levels when albuminuria is present. This study reveals specific links between immune response and controlled hypertension in patients who develop albuminuria, pointing to potential protein targets for novel and future therapeutic interventions.

## Introduction

Urinary albumin excretion is an indicator of worse cardiovascular prognosis, independently of renal function or diabetes^1–3^. Particularly in hypertensives, clinical guidelines recommend the use of albuminuria to assess target organ damage^4^, and recent studies point to its value not only in cardiovascular (CV) risk stratification but also in predicting individual prognosis or assessing therapeutic efficiency^5^. The ability of renin-angiotensin system (RAS) suppression to control blood pressure in hypertensive individuals has been amply demonstrated^6–8^. However, recent studies conducted by our group and others showed high albuminuria development in a relevant percentage of patients undergoing chronic RAS suppression, either as maintained (MHA) or as *de novo* developed albuminuria (dnA)^5,9^. These patients are likely at the highest risk of progression of CV and renal disease. The reason why some of these controlled hypertensive individuals develop albuminuria and others do not, persistant normoalbuminuric patients (N), is uncertain. Subjacent physiopathological alterations are unknown and this phenomenon is difficult to predict and avoid.

Immune response was linked to hypertension, together with inflammation, far beyond CV disease. Participation of immune cells in hypertension was demonstrated and also the efficiency of immunosuppressants in BP diminishment in animal models^10,11^. Vaccination was even tentatively proposed as a future therapy for prevention^12,13^. Previous findings point to the immune system in modulation of renal injury^14^. In particular, an increased number of infiltrating immune cells was observed in this organ in experimental hypertensive models^15^. Our group recently identified urinary CD59, an inhibitor of the membrane attack complex (MAC) to protect cells in an inflammatory scenario, and a sub-set of plasma immunoglobulins as key molecules behind physiopathological mechanisms responsible for albuminuria development in hypertensive patients under chronic RAS suppression^16,17^.

Urine composition not only reflects normal kidney function but also contains specific proteins produced in the kidney which may be altered in response to CV disease^18^, diabetic nephropathy^19^, and chronic kidney disease^20^, among others^21^. Here, we pursued the identification of protein indicators detectable in urine linked to albuminuria development in hypertensive patients under chronic RAS suppression. For such purpose, we screened the urinary proteome widely by using a non-biased (non-target) quantitative approach based on peptide labelling (iTRAQ methodology) and highly sensitive mass spectrometry detection (MS).

## Results

**Figure 1:**
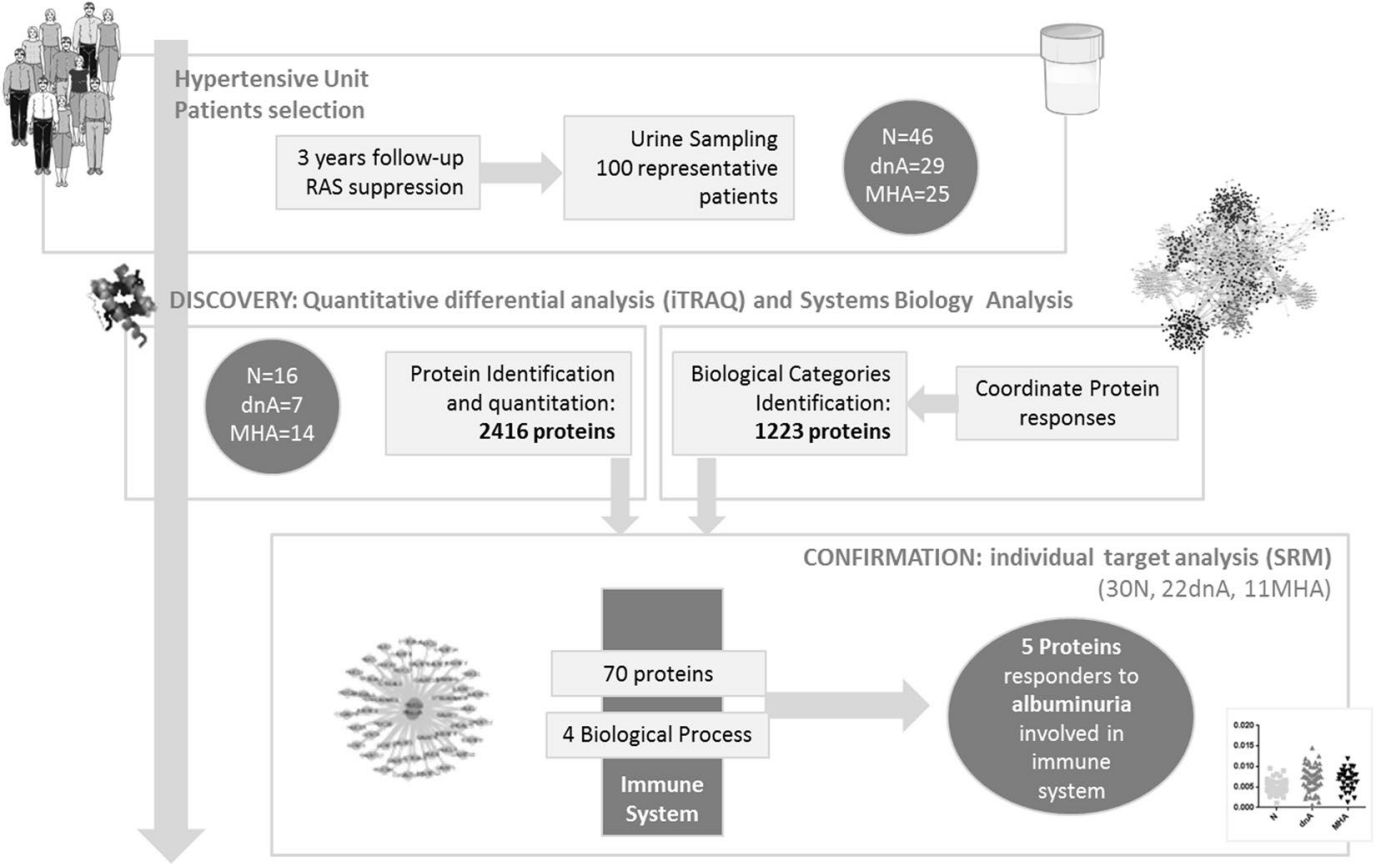
Schematic workflow showing the different parts of the study from patients selection to discovery and confirmation stages.

This study extends our previous knowledge in the search for urinary protein alterations in hypertensive patients who develop albuminuria despite of chronic RAS suppression. Continuous development of proteomic tools allows to widely cover the urinome and detect differences otherwise hidden. In this sense, a highly-sensitive and quantitative protein analysis has been performed in urine in this clinical setting. Urinary changes were investigated by a double approach: 1) shotgun data revealed individual proteins with altered levels in albuminuric patients (dnA or MHA *vs* N); and 2) systems biology analysis revealed biological processes (functional categories) altered as the result of coordinated protein responses (Supplementary Figure 1). Identification of significant alterations was approached as detailed in methods section for both, individual proteins and biological processes. A detailed workflow is shown in Fig. 1. All proteomics data derived from this study are deposited in Peptide Atlas and are accessible through the accession number PASS01069.

### Immune response: a main alteration in hypertensives under chronic RAS suppression developing albuminuria

In the shotgun experiment, a total of 2416 proteins were identified (Supplementary Table 1). Albumin was identified with the expected increase in dnA and MHA *vs* N. CD59 and alpha-1 antitrypsin also confirmed their variation in urine of albuminuric hypertensives under chronic RAS suppression as previously published by our group^17^. A total of 1223 categories (biological processes) were identified (Supplementary Table 2). When this double approach was merged, four categories related to immune response highlight: “immune response of cells”, “adhesion of immune cells”, “adhesion of blood cells” and “phagocytosis” (Table 1). A total of 70 unique proteins compose these functional categories (see Supplementary Tables 3–6 showing individual protein responses). As expected, some proteins are shared by various categories. In particular, 23 proteins are categorized in immune response of cells in albuminuric individuals. Interaction networks analysis relate them with specific molecular processes. In particular, ANXA1, CLEC7A, DOCK2, and CD4 are involved in T-cells activation and differentiation; dendritic cells regulation involves GAS6 and PRTN3; and ANXA1, AZU1, C3, GAS6, CSF1, CD14, CD4 and THBS1 participate in cytokine production and regulation.

**Table 1:** Immune response categories significantly altered in hypertensive patients under chronic RAS suppression who develop albuminuria.

Biological Process	dnA/N			MHA/N		
	Z	FDR	Total proteins	Z	FDR	Total proteins
Adhesion of blood cells	−3.44	0.03	55	−2.97	0.09	55
Adhesion of immune cells	−3.40	0.03	45	−3.03	0.08	45
Immune response of cells	−3.47	0.03	23	−3.54	0.02	23
Phagocytosis	−3.40	0.03	25	−3.56	0.02	25

A negative Z value indicates down-regulation in dnA or MHA versus N. The number of proteins included in each biological process (categories) is also indicated. N: normoalbuminuria; dnA: de novo albuminuria; MHA: maintained high albuminuria.

Adhesive interactions mediate migration of cells to sites of inflammation. Adhesion is mediated by specific molecules, called adhesion molecules, which are classified mainly in terms of their dependence of calcium. Adhesion of immune cells and blood cells categories content 45 and 55 proteins, respectively. Regarding to calcium independent proteins, the MAC (membrane attack complex) proteins: MCAM, ICAM, PECAM and VCAM showed reduced urinary levels in albuminuric patients. VCAM1 mediates the adhesion of lymphocytes, monocytes, eosinophils and basophils while ICAM1 do the same for inflammatory immune cells as macrophages and granulocytes. Both of them cluster with activated moesin and ezrin^22^, which were found here with contrary trend to the MAC proteins (increased levels in albuminuric individuals).

**Figure 2:**
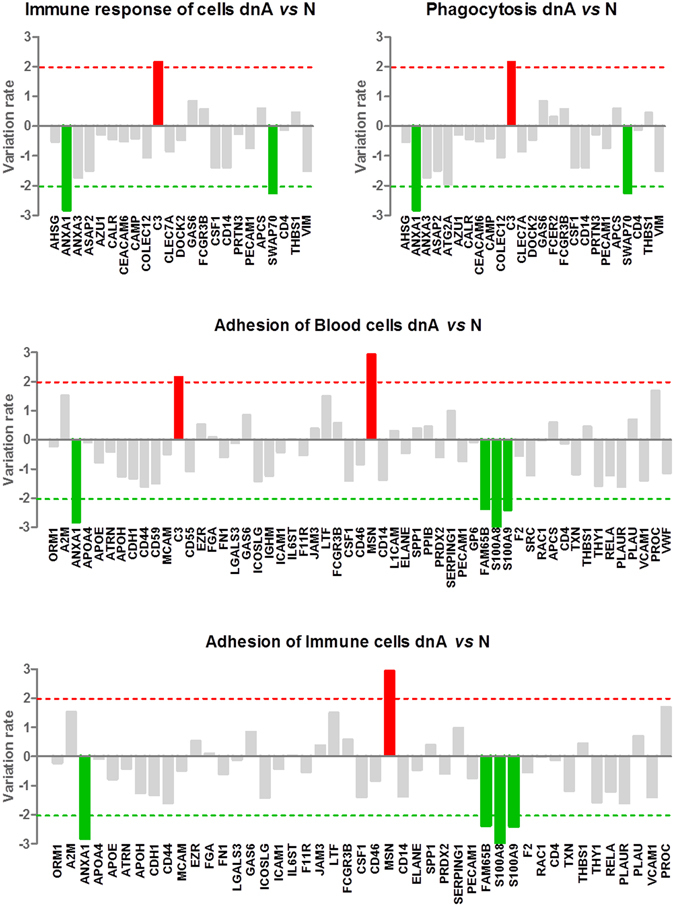
Functional categories related to immune response showing significant alteration between dnA and N (Z value ≥ |2|, FDR < 0.05). Variation rates for each individual protein are shown. dnA: *de novo* albuminuria, N: normoalbuminuria.

**Figure 3:**
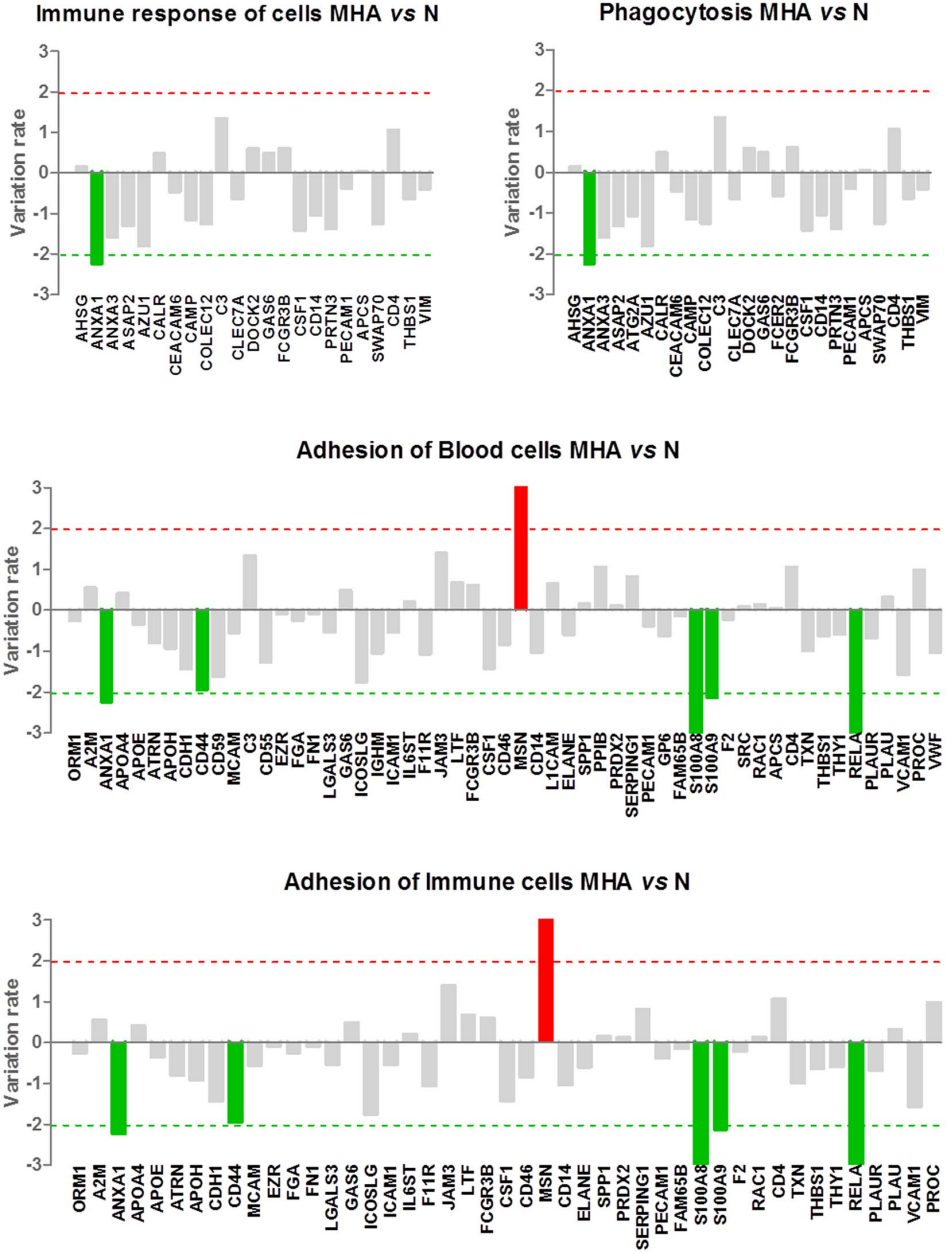
Functional categories related to immune response showing significant alteration between MHA and N (Z value ≥ |2|, FDR < 0.05). Variation rates for each individual protein are shown. MHA: maintained high albuminuria, N: normoalbuminuria.

Phagocytosis is an essential part of the immune response, both, innate and adaptive, also involved in tissue remodeling and homeostasis^23^. Phagocytosis promotes the release of pro-inflammatory cytokines^24^ and 25 proteins were here identified in urine linked to this process. Mainly, binding proteins as CAMP, CLEC7A, CSF1, CALR, DOCK2, SWAP70, ASAP2, C3 and GAS6; and proteins with catalytic activity as CAMP, DOCK2, AZU, AHSG, ASAP2, PRTN3 and C3. Figures 2 and 3 represent the 70 proteins and their role in immune response. Protein variation rate in dnA or MHA compared to N are represented.

### Specific protein signatures reveal immune deregulation

**Figure 4:**
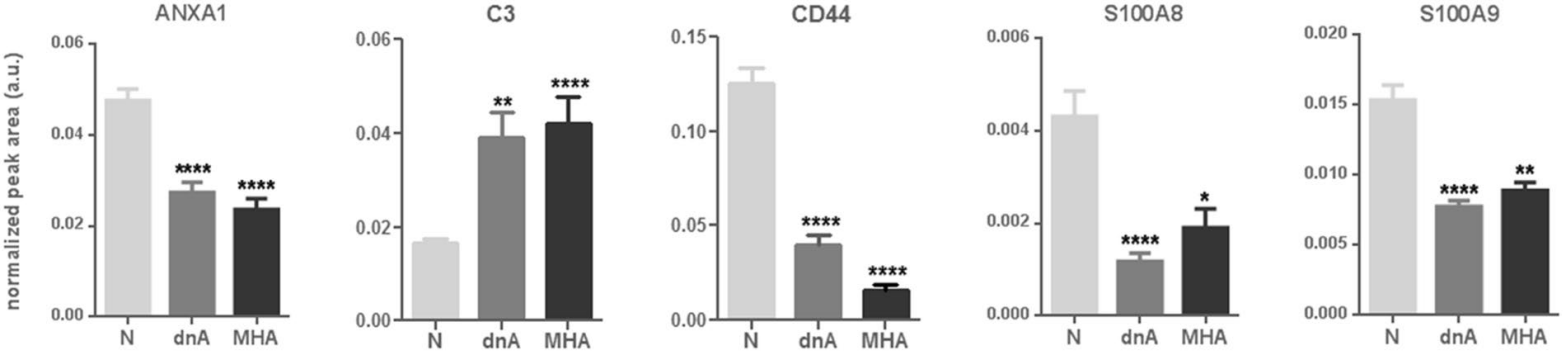
ANXA1, C3, CD44, S100A8 and S100A9 showed significantly altered levels in urine from albuminuric patients by SRM-LC-MS/MS. Mann-Whitney test (95% confidence level) was applied.

**Figure 5:**
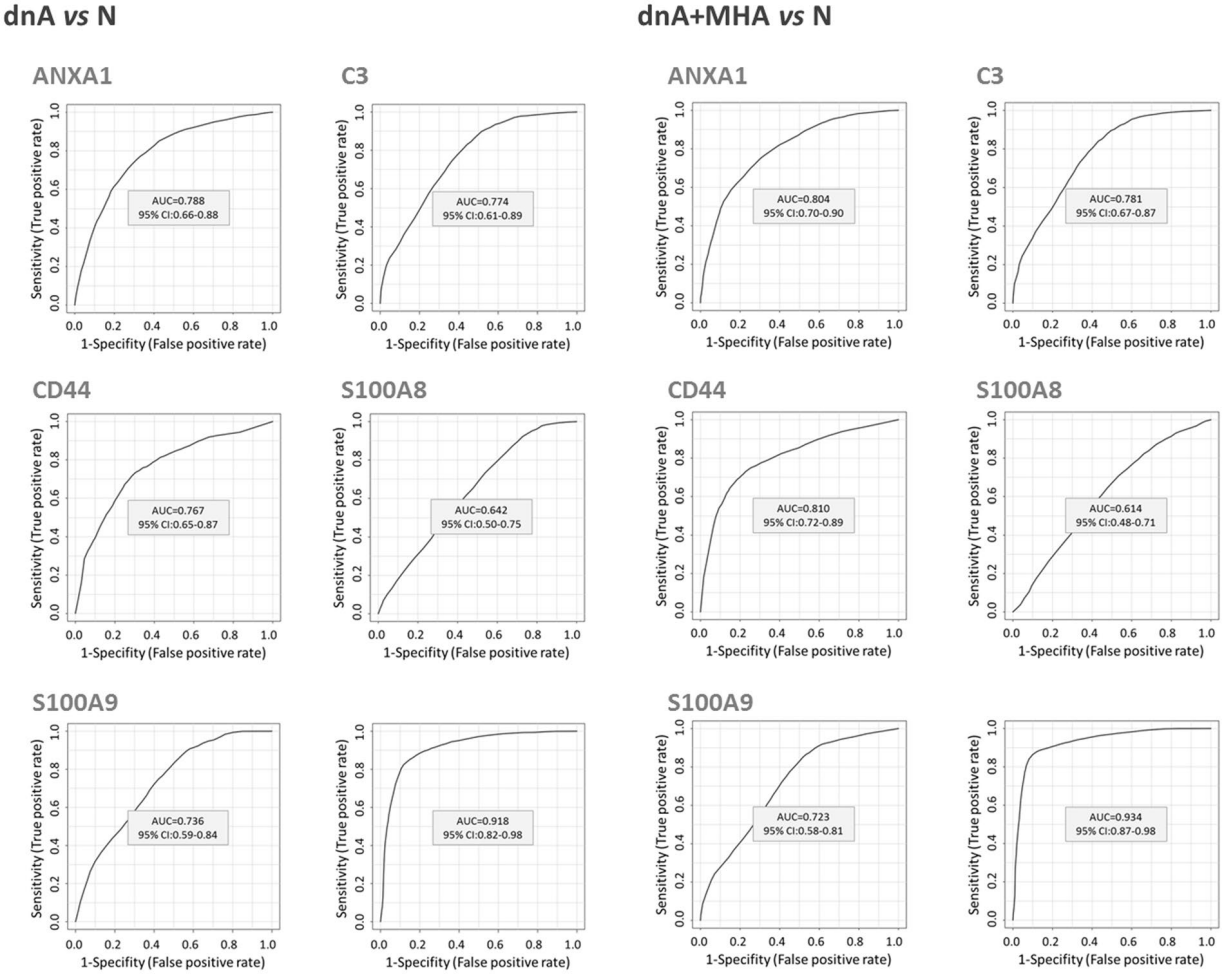
Receiver operating curves (ROC) for the five confirmed proteins are shown, individually and combined. Responses were evaluated by analyzing N *vs* dnA, and N *vs* dnA + MHA. N: normoalbuminuria, dnA: *de novo* albuminuria, MHA: maintained high albuminuria.

**Figure 6:**
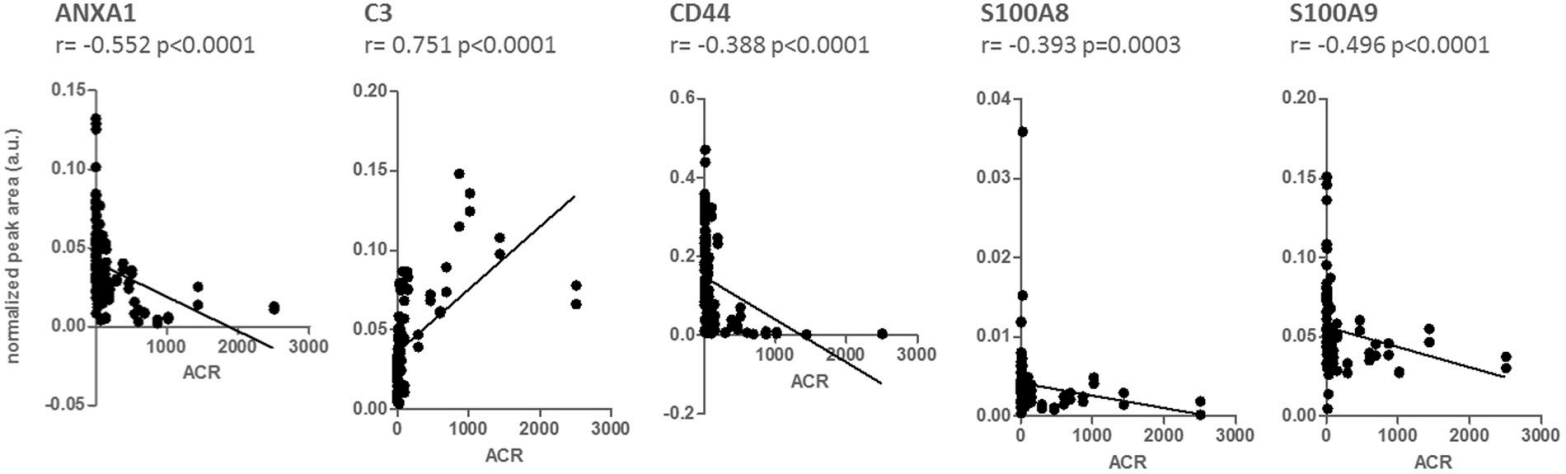
Graphs show correlations (Spearman correlation) between confirmed proteins and ACR (albumin to creatinine ratio).

Individual variation of immune system proteins in response to albuminuria development was evaluated and five proteins were selected with significant variation in dnA and MHA compared to N (variation rate ≥ |2| and a minimum of 3 peptides identified) (Table 2). Significantly altered proteins are: the complement system protein C3, found increased, and Annexin A1 (ANXA1), S100A8, S100A9, and the glycoprotein CD44, found decreased. In particular, adhesion of blood cells comprises ANXA1, CD44, C3, S100A8 and S100A9; adhesion of immune cells comprises ANXA1, CD44, S100A8 and S100A9; and immune response of cells and phagocytosis includes ANXA1 and C3 (Figs 2 and 3). These five proteins were confirmed by target mass spectrometry (SRM-LC-MS/MS) in an independent cohort of 63 hypertensive individuals as shown in Fig. 4 (Supplementary Table 7). Receiver operating curves (ROC) are shown in Fig. 5. Individual ROC curves show good sensitivity and specificity which is considerably improved when individual responses are combined (see also Supplementary Figure 2). Correlations with albuminuria were also calculated showing significant correlation in all cases (Fig. 6).

**Table 2:** Proteins with significant variation in urine by iTRAQ analysis in hypertensives under chronic RAS suppression developing albuminuria.

**Protein**	Uniprot	Gene	ID peptides	Variation rate	
				dnA/N	MHA/N
***Annexin A1***	P04083	ANXA1	15	−2.8	−2.2
***CD44 antigen***	P16070	CD44	9	−1.6	−1.9
***Complement C3***	P01024	C3	77	2.2	1.3
***Protein S100-A8***	P05109	S100A8	20	−3.3	−3.1
***Protein S100-A9***	P06702	S100A9	16	−2.4	−2.1

Variation rate is expressed as the difference in average Zq values between groups, for the two comparisons (dnA/N and MHA/N). Zq values: log2-ratios expressed in form of the standardized variables. N: Normoalbuminuria; dnA: de novo albuminuria; MHA: maintained high albuminuria.

## Discussion

One of the main challenges in the management of hypertensive patients under chronic suppression of RAS is understanding why some of them develop albuminuria and thus have a worse prognosis. The link between hypertension and immune system has been revised^25^, mainly based on models of genetic hypertension (spontaneously hypertensive rats, SHR), induced hypertension with DOCA/salt, Angiotensin II or renal damage. Here we applied a non-target analysis combined with a recenlty developed algorithm designed for system biology analysis. In this way, we could achieve a wide coverage of the urinary proteome and identify coordinated protein behaviour in response to albuminuria development in hypertensives under RAS suppression. Immune response alteration was identified in terms of immune response of cells, phagocytosis, and cell adhesion. In particular, main alterations were identified for the proteins C3, CD44, ANXA1, S100A8 and S100A9.

The cell mediated immunity involves activation of T-lymphocytes, macrophages and natural killer cells, and the release of cytokines in response to an antigen. Alterations of such immune response here detectable in urine comprise 23 proteins (Figs 2 and 3). Among them, the two most contributing are C3 and ANXA1. C3 levels are increased in plasma during hypertension development^26^ and it is even higher in patients with resistant hypertension^27^. High levels of C3 correlated with higher risk of developing hypertension, diabetes or myocardial infarction^28^. Our findings are also consistent with a local synthesis of C3 from renal epithelial cells in proteinuria-associated renal disease^29^. In this sense, C3 deposits were found in proximal tubular cells in kidneys of proteinuric patients^30^, in agreement with an excess protein traffic occurring in proteinuric nephropathies, causing an inflammatory response with local increase of C3^31^. Reduced expression of ANXA1 was observed in aortas from MgL mice with low intracellular magnesium levels (and high blood pressure)^32^. ANXA1 is an endogenous repressor of inflammation and increased urinary levels have been linked to glomerular injury. However, our patients do exhibit mildly decreased eGFR, thus observed reduced levels in urine may suggest an impaired compensation of inflammation by ANXA1 in albuminuric patients. C3 and ANXA1 also play a key role in phagocytosis in different cells and diseases, such as the phagocytosis of apoptotic leukocytes during peripheral inflammation^33^ or the macrophage mediated phagocytosis of apoptotic cells for clearance^34^. Here we show clear de-regulation of these proteins in response to albuminuria development.

Cell adhesion was also shown to be deregulated. Adhesive interactions mediate migration of cells to sites of inflammation. In particular, CD44 is a calcium-independent adhesion molecule that participates in lymphocyte activation. Recent studies propose that high levels of AngII promote an increase in circulating levels of CD44^35^, and that inhibiting CD44 interaction with hyaluronic acid would be useful as therapeutic target by reducing the negative CV effects of Ang II^36^. Here, we found a reduction in CD44 urine levels in albuminuric patients RAS suppressed.

Finally, S100A8 and S100A9 are two calcium-binding proteins that constitute a heterodimer called calprotectin with high affinity not only for calcium but also for zinc and magnesium, playing an important role in the metal availability in immune system. Increased levels were found in cardiac tissue microarrays, heart and heart neutrophils when AngII is infused^37^. S100A8 was severely diminished in monocytes from acute coronary syndrome patients compared to healthy controls^38^. These two proteins have different roles as scavenging reactive oxygen species or contribution to phagocytosis^39^. In particular, calprotectin inhibits MMPs whose activity was found increased previously by our group in this clinical scenario in agreement with reduced levels observed here for these two proteins^40^.

Apart from these main protein alterations, when looking for the interactions among the 70 unique proteins identified related to immune response, 41 proteins are interconnected (see the immune network behind albuminuria development in Supplementary Figure 3). Fourteen proteins substantially varied already in dnA (earlier response), constituting a molecular fingerprint which deserves particular attention for further confirmation in subsequent studies. Particularly, CD59 was already identified in a previous study from our group with the same trend by a different technical approach. We found vimentin altered in the intima and media layers from atherosclerotic coronary pointing to cytoskeleton disassembly^41,42^. APOH was pointed as a potential clinical marker of cardiovascular disease^43^. Thioredoxin plays a role in cellular defense against oxidative stress in cardiovascular disease^44^ and reverses age-related hypertension attenuating endothelial dysfunction and increasing nitric oxide production^45^.

Albuminuria is currently the key indicator to follow for an early intervention in these hypertensive patients who are under chronic RAS suppression, aimed to prevent from chronic kidney disease (CKD) development. Previously, we identified specific proteins in plasma associated with albuminuria development which point to the activation of the immune system^16^. This study widely covers the urinary proteome in the context of hypertensive patients who develop albuminuria despite of chronic RAS suppression. Main subjacent alterations in terms of biological processes underlying albuminuria development involve immune cells and immune response of cells. Thus, we further confirm the link between immune system and hypertension. Specific protein signatures linked to albuminuria development and to be targeted in future therapeutic interventions have been confirmed, and further studies should follow to investigate additional proteins of potential interest. The role of immune system in this clinical context is here clearly manifested. As main weakness to be acknowledged is the limited number of patients. The number of recruited individuals should be increased in future studies to confirm the potential value of the protein indicators here described.

Related to the coverage of the urinary proteome achieved in this study, it is important to note that the main purpose was the identification of those proteins showing alteration in this clinical context. Knowing that, we compared our data with the “human urine” repository of PeptideAtlas, finding that 69% of the proteins identified here were not included in such repository and are thus complementary.

In conclusion, urine reflects molecular changes linked to albuminuria development in hypertensive patients with controlled blood pressure and novel clues in the association between immune response and hypertension are shown here. The silent and hidden albuminuria development is of special interest in the search of a better stratification of patients at risk so that a better preventive treatment could be designed and applied earlier. The molecular signature here identified in urine points to specific protein targets for novel therapeutic interventions in the management of hypertensives.

## Methods

### Patient selection and urine collection

Patient selection was based on a previous study showing the development of *de novo* albuminuria in patients during chronic RAS suppression^9^ and performed as described before^46^. Briefly, patients from the Hypertension Unit-Hospital 12 de Octubre were followed-up for a minimum of three years. At the end of that period patients were classified as follows: a) patients who remained normoalbuminuric during the follow-up (N); b) patients developing *de novo* albuminuria during the follow-up (dnA); c) patients with maintained albuminuria since baseline and during the follow-up (MHA). Urine was collected at the end of the follow-up. A total of 100 hypertensives under RAS suppression, representative of the three groups, were included (46N, 29dnA and 25MHA). The study was approved by the Ethics Committee of the Hospital 12 de Octubre and was conducted according to the principles of the Declaration of Helsinki. All patients signed written informed consent before inclusion. N, dnA and MHA patients’ groups were compared by One-way ANOVA to identify significant differences in clinical characteristics or medication (Table 3). Urine samples were processed and stored as described before^17,18^.

**Table 3:** Clinical characteristics of hypertensive patients under chronic RAS suppression included in the discovery (iTRAQ) and confirmation (SRM) cohorts.

	Discovery Cohort				Confirmation Cohort			
	N (n = 16)	dnA (n = 7)	MHA (n = 14)	P *value*	N (n = 30)	dnA (n = 22)	MHA (n = 11)	P *value*
Age, y	63 ± 13	61 ± 9	60 ± 8	0.685	63 ± 10	68 ± 7	68 ± 10	0.078
Male sex, %	38	71	57	0.298	37	64	64	0.104
Current smoking, %	12	29	21	0.654	13	18	18	0.876
Diabetes, %	38	14	50	0.300	17	55	28	0.013*
Systolic blood pressure, mmHg	132 ± 14	135 ± 8	133 ± 18	0.879	139 ± 18	137 ± 21	137 ± 37	0.968
Diastolic blood pressure, mmHg	79 ± 13	83 ± 9	82 ± 10	0.705	82 ± 10	81 ± 10	77 ± 20	0.445
BMI, kg/m^2^	31 ± 5	29 ± 2	32 ± 5	0.344	30 ± 4	30 ± 5	29 ± 4	0.858
Albumin/creatinine, mg/g	9 ± 9	46 ± 38	72 ± 94	0.026*	6 ± 14	127 ± 198	374 ± 45	<0.0001*
Creatinine clearance rate, mg/mL	112 ± 39	114 ± 53	99 ± 34	0.724	195 ± 467	88 ± 45	59 ± 22	0.388
eGFR, mL/min/1.73 m^2^	83 ± 17	85 ± 23	78 ± 25	0.700	84 ± 16	67 ± 19	56 ± 21	<0.0001*
Total cholesterol, mg/dL	194 ± 30	158 ± 13	170 ± 20	0.004*	185 ± 32	165 ± 27	172 ± 32	0.062
Triglycerides, mg/dL	106 ± 50	124 ± 71	149 ± 87	0.249	122 ± 46	131 ± 64	99 ± 28	0.247
HDL cholesterol, mg/dL	57 ± 12	50 ± 19	44 ± 13	0.078	54 ± 13	49 ± 10	52 ± 14	0.420
LDL cholesterol, mg/dL	115 ± 28	83 ± 13	98 ± 17	0.008*	107 ± 31	89 ± 18	100 ± 23	0.050
Glycemia, mg/dL	103 ± 29	105 ± 12	109 ± 27	0.834	109 ± 25	120 ± 24	120 ± 42	0.305
Uric acid, mg/dL	5 ± 1	6 ± 2	7 ± 2	0.034*	5 ± 2	6 ± 2	7 ± 2	0.008*
ACEi, %	19	0	29	0.307	10	23	9	0.385
ARB, %	75	86	57	0.366	80	68	82	0.559
Diuretic, %	50	71	64	0.586	57	45	36	0.481
Calcium channel blocker, %	62	29	50	0.342	53	59	82	0.262
β blocker agent, %	19	14	36	0.463	30	32	18	0.706
α blocker agent, %	19	29	14	0.750	10	32	27	0.136
Anticoagulant, %	6	0	0	0.532	3	0	9	0.384
Lipid-lowering agent, %	69	57	86	0.355	80	82	64	0.470
Antidiabetic agent, %	31	14	43	0.438	10	36	18	0.068
Anti aldosteronics, %	0	14	29	0.075	17	18	9	0.795
Hypouricemic Agent, %	12	14	14	0.989	0	18	9	0.056

Values are expressed as mean ± SD or percentage (%). N: normoalbuminuria; dnA: de novo albuminuria; MHA: maintained high albuminuria.

### Quantitative iTRAQ-LC-MS/MS differential analysis

#### Protein digestion and isobaric labelling

Urine samples from a total of 37 hypertensive individuals under chronic RAS suppression were used for the quantitative differential analysis by liquid chromatography coupled to mass spectrometry in tandem (LC-MS/MS) (discovery phase). Isobaric tags for relative and absolute quantification experiment (iTRAQ, 8-plex) was performed as previously published^16^. Four biological replicates were analyzed per condition (N, dnA, MHA), combining aliquots of 1–4 samples per replicate. 150 μg of total protein from each biological condition were loaded in SDS-PAGE gels to concentrate all the proteins in a single band. Proteins were digested using the in-gel digestion protocol as previously described^47^. Briefly, gel bands were reduced with 10 mM DTT, alkylated in 50 mM iodoacetamide, and digested overnight at 37 C with 30 ng/mL modified trypsin (Promega, Madison, WI, USA) at 12:1 protein:trypsin (w/w) ratio in 50 mM ammonium bicarbonate, pH 8.8 containing 10% acetonitrile. The resulting tryptic peptides were extracted by incubation in 12 mM ammonium bicarbonate pH 8.8 and later, 0.5% trifluoroacetic acid (TFA). TFA was added to a final concentration of 1% and the peptides were desalted onto C18 Oasis-HLB cartridges and dried-down for further analysis. For stable isobaric labelling, the resulting tryptic peptides were dissolved in 100 mM Triethylammonium bicarbonate (TEAB) buffer, and the peptide concentration was determined by measuring amide bonds with the Direct Detect system (Millipore). Equal amounts of each peptide sample were labelled using the 8-plex iTRAQ Reagents Multiplex Kit (Applied Biosystems, Foster City, CA, USA) according to manufacturer’s protocol. Normoalbuminuric, dnA and MHA samples were labelled with iTRAQ reagents previously reconstituted with 70 μl of isopropanol. After incubation at room temperature for 2 h, reaction was stopped with 0.5% TFA, incubated for 30 min and peptides were combined. Samples were concentrated in a Speed Vac, desalted onto C18 Oasis-HLB cartridges and dried-down for further analysis. For increasing proteome coverage, iTRAQ-labelled samples were fractionated by mixed-cation exchange chromatography (Oasis HLB-MCX columns) into six fractions, desalted and concentrated as before.

#### Protein identification and quantitation

Labelled peptides were analyzed by LC-MS/MS using a C-18 reversed phase nano-column (75 µm I.D. × 50 cm, 2 µm particle size, Acclaim PepMap RSLC, 100 C18; Thermo Fisher Scientific, Waltham, MA, USA) in a continuous acetonitrile gradient consisting of 0–30% B in 360 min, 50–90% B in 3 min (A = 0.1% formic acid; B = 90% acetonitrile, 0.1% formic acid). A flow rate of 200 nL/min was used to elute peptides from the nano-column to an emitter nanospray needle for real time ionization and peptide fragmentation on a Q-Exactive mass spectrometer (Thermo Fisher). An enhanced FT-resolution spectrum (resolution = 70,000) followed by the MS/MS spectra from the 15 most intense parent ions were analyzed along the chromatographic run. Dynamic exclusion was set at 40 s. For peptide identification, all spectra were analyzed with Proteome Discoverer (version 1.4.0.29, Thermo Fisher Scientific) using SEQUEST-HT (Thermo Fisher Scientific). For database searching at the Uniprot database containing all sequences from human and crap contaminants (March 06, 2013; 70024 entries), the parameters were selected as follows: trypsin digestion with 2 maximum missed cleavage sites, precursor and fragment mass tolerances of 2 Da and 0.02 Da, respectively, carbamidomethyl cysteine, iTRAQ modifications at N-terminal and Lys residues as fixed modifications, and methionine oxidation as dynamic modification. Peptide identification was validated using the probability ratio method^48^. False discovery rate (FDR) was calculated using inverted databases, and the refined method^49^ with an additional filtering for precursor mass tolerance of 12 ppm^50^. Only peptides with a confidence of at least 95% were used to quantify the relative abundance of each peptide determined as previously described. Protein quantification from reporter ion intensities and statistical analysis of quantitative data to identify significant proteins were performed using QuiXoT based on a statistical model previously described^51^. In this model protein log2-ratios are expressed in form of the standardized variables, i.e., in units of standard deviation according to their estimated variances (Zq values). Individual protein responses to albuminuria development were evaluated. An average of Zq value of the four biological replicates was calculated per condition. Ratio of change between two conditions was expressed as the difference in average Zq values between compared groups (dnA/N and MHA/N).Those proteins with ratio of change between groups ≥|2|, and a minimum of 3 peptides identified were selected for further analysis.

### System biology analysis

A system biology analysis of the whole set of identified proteins was performed using a novel algorithm developed specifically for the analysis of coordinated protein responses in high-throughput quantitative proteomics experiments^52^. In this way, changes in functional biological processes produced by the coordinated protein behavior can be detected far beyond individual protein responses.

Briefly, this algorithm correlates the performance of a group of proteins inside of a category (biological process) in terms of their quantitative behavior (relative abundance). As a result of this coordinated behavior, a Z value is assigned to each category together with a FDR rate. Categories including at least five proteins were considered. To identify the significant biological process altered in chronically treated hypertensive patients, dnA and MHA groups were compared to the N group applying restricted parameters and selecting those categories with a Z value ≥ |2| and FDR < 0.05. Protein pathway analysis was performed for the proteins identified in those categories of interest by STRING 9.0^53^ consisting of a database of known and predicted protein interactions, including direct (physical) and indirect (functional) associations derived from genomics, high-throughput experiments and coexpression. High confidence filter (0.900) was applied.

### Target protein analysis (Selected Reaction Monitoring (SRM)-LC-MS/MS)

Urine samples from 63 hypertensive patients under chronic RAS suppression were analyzed, individually (not pools), by label-free SRM-LC-MS/MS (selected reaction monitoring) as previously published (see Supplementary Table 8 for details)^17,18,54,55^. Briefly, in-silico digestion of the protein of interest was done with Skyline software. Proteotypic peptides (confirmed by BLAST) were selected and analysis conditions were optimized by means of Skyline software. Transition masses (precursor ion and product ion), collision energy and fragmentor potential are then selected for confirmation analysis.Tryptic digest from 30 µg total protein were analyzed in a 6460 Triple Quadrupole LC/MS/MS on-line connected to nanoLC in a Chip-format configuration (ChipCube interface, Agilent Technologies) and 1200 Series LC Modules (Agilent Technologies). Peptide separation was carried out onto a ProtID Zorbax 300B-C18-5 μm chip with 43 × 0.075-mm analytical column and 40 nL enrichment column (Agilent Technologies). Two microliters of sample was injected at 3 μL/min and separation took place at 0.4 μL/min as follow: 1) 0–3 min 5% B, 2) At 10 min 70% B, 3) At 12 min 95% B, 4) At 14 min 95% B, 5) 14.2–15 min 5% B (A = 0.1% formic acid in double-distilled water; B = 0.1% formic acid in acetonitrile). The fragmentor was set to 130 V, dwell time to 20 ms, delta EMV to 600 V, and collision energy was optimized for each SRM transition. The system was controlled by Mass Hunter LC–MS Acquisition Software (v4.01). Individual signals were normalized based on TIC (total ion current) and normalized peak areas were calculated for inter-group comparison. Statistical analyses were performed by means of GraphPad Prism 6 (version 6.01) software. The ROUT method was applied to detect outliers based on the FDR, setting Q value to 5%. Mann-Whitney non-parametric test (95% confidence level) was performed. ROC curves were obtained considering all transitions for every protein (Supplementary Table 8) with Metabolanalyst software using ROC curve based model evaluation (Tester) and Random forest algorithm. Proteins were evaluated separately and combined, for the two comparisons: N vs dnA and N vs dnA + MHA.

## Electronic supplementary material

Supplementary Figures

Supplementary Tables
