# Author Correction: Immune system deregulation in hypertensive patients chronically RAS suppressed developing albuminuria

**DOI:** 10.1038/s41598-018-22185-0

**Published:** 2018-03-02

**Authors:** Marta Martin-Lorenzo, Laura Gonzalez-Calero, Paula J. Martinez, Montserrat Baldan-Martin, Juan Antonio Lopez, Gema Ruiz-Hurtado, Fernando de la Cuesta, Julián Segura, Jesús Vazquez, Fernando Vivanco, Maria G. Barderas, Luis M. Ruilope, Gloria Alvarez-Llamas

**Affiliations:** 1 Departament of Immunology, IIS-Fundacion JimenezDiaz, REDinREN, Madrid, Spain; 2 Department of Vascular Physiopathology, Hospital Nacional de Paraplejicos SESCAM, Toledo, Spain; 3 Laboratory of Cardiovascular Proteomics CNIC, Madrid, Spain; 4 Hypertension Unit, Instituto de Investigación imas12, Hospital Universitario 12 de Octubre, Madrid, Spain; 5 Department of Biochemistry and Molecular Biology I, Universidad Complutense, Madrid, Spain; 6 Universidad Europea, Madrid, Spain

Correction to: *Scientific Reports* 10.1038/s41598-017-09042-2, published online 21 August 2017

The Acknowledgements section in this Article is incomplete.

“The authors acknowledge Lucía Guerrero and Maria Cruz Casal from Hospital 12 de Octubre for their participation in samples collection. Instituto de Salud Carlos III co- supported by FEDER grants (PI11/01401, PI13/01873, PI14/01650, PI16/01334, CPII15/00027, PT13/0001/0013, IF08/3667-1, PI11/02239, PI11/02432, PI14/0917, PI14/01841, PIE13/00045, CP15/00129, PI13/01746, REDinREN (RD12/0021/0001, RD12/0042/0071 and RD16/0009)), and Fundación SENEFRO. These results are lined up with the Spanish initiative on the Human Proteome Project (SpHPP).”

should read:

“The authors acknowledge Lucía Guerrero and Maria Cruz Casal from Hospital 12 de Octubre for their participation in samples collection. Instituto de Salud Carlos III co- supported by FEDER grants (PI11/01401, PI13/01873, PI14/01650, PI16/01334, CPII15/00027, PT13/0001/0013, IF08/3667-1, PI11/02239, PI11/02432, PI14/0917, PI14/01841, PIE13/00045, CP15/00129, PI13/01746, REDinREN (RD12/0021/0001, RD12/0042/0071 and RD16/0009)), and Fundación SENEFRO. JAL and JV belong to CIBER de Enfermedades Cardiovasculares (CIBERCV), Madrid, Spain. These results are lined up with the Spanish initiative on the Human Proteome Project (SpHPP).”

