# Supplementary material for: Immune system deregulation in hypertensive patients chronically RAS suppressed developing albuminuria
Source: Sci Rep. 2017 Aug 21;7:8894. doi: 10.1038/s41598-017-09042-2 (PMC5566220; doi:10.1038/s41598-017-09042-2)
Supplement: Supplementary file 1 — Supplementary Figures [file 41598_2017_9042_MOESM1_ESM.pdf]

Immune system deregulation in hypertensive patients chronically RAS suppressed developing albuminuria

Marta Martin-Lorenzo[1]#, Laura Gonzalez-Calero[1]#, Paula J Martinez[1], Montserrat Baldan-Martin[2], Juan Antonio Lopez[3], Gema Ruiz-Hurtado[4], Fernando de la Cuesta[2], Julián Segura[4], Jesús Vazquez[3], Fernando Vivanco[1,5], Maria G Barderas[2], Luis M Ruilope[4,6*], Gloria Alvarez-Llamas[1*]

[1]Departament of Immunology. IIS-Fundacion JimenezDiaz, REDinREN, Madrid. Spain
[2]Department of Vascular Physiopathology, Hospital Nacional de Paraplejicos SESCAM, Toledo. Spain
[3]Laboratory of Cardiovascular Proteomics CNIC, Madrid, Spain.
[4]Hypertension Unit, Instituto de Investigación imas12, Hospital Universitario 12 de Octubre, Madrid, Spain.
[5]Departamento de Bioquimica y Biologia Molecular I, Universidad Complutense, Madrid, Spain.
[6]Universidad Europea, Madrid, Spain.

#Both authors contributed equally to this work

[*]To whom correspondence should be addressed:

Gloria Alvarez-Llamas. IIS-Fundación Jiménez Díaz. Avenida Reyes Católicos 2, 28040 Madrid. Phone N. +34 915504800 ext.2202. Fax N. +34 915448246. email: galvarez@fjd.es

Luis M Ruilope, Unidad de Hipertension, Instituto de Investigación i+12, Hospital Universitario 12 de Octubre, Madrid, Spain. Telephone: +34 914317741. FAX: +34915765644. email: ruilope@ad-hocbox.com.

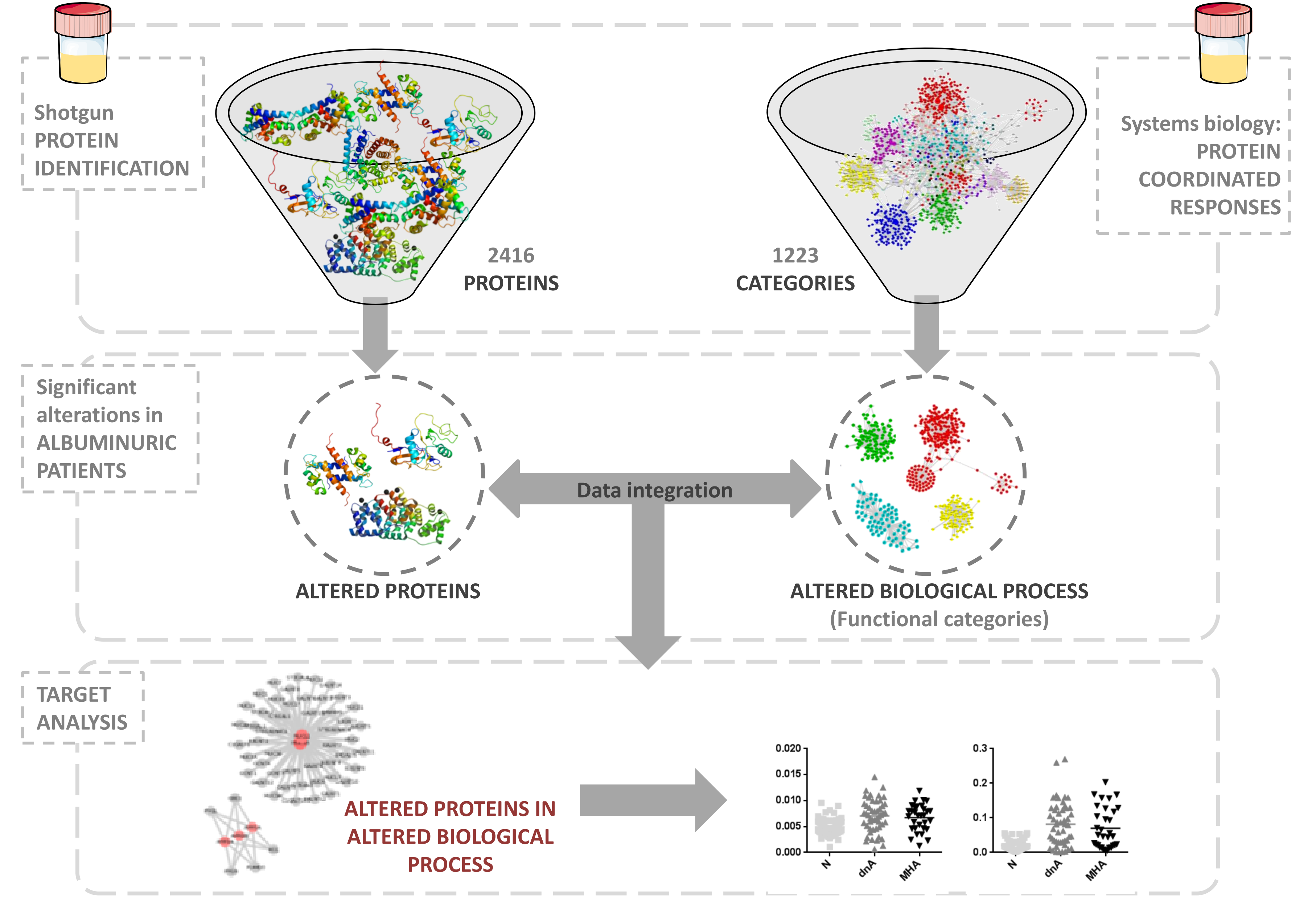

Supplementary Figure1. A total of 2416 proteins were identified (LC-MS/MS, shotgun experiment). Quantitative analysis revealed proteins showing altered levels in urine from albuminuric hypertensive patients (iTRAQ). In parallel, 1223 functional categories were found based on protein coordinated responses, and biological processes siginificantly altered were identified. Highlighted proteins and biological processes were matched, pointing to a role for the immune system. Selected proteins variation was confirmed by target analysis (SRM-LC-MS/MS).

N vs dnA

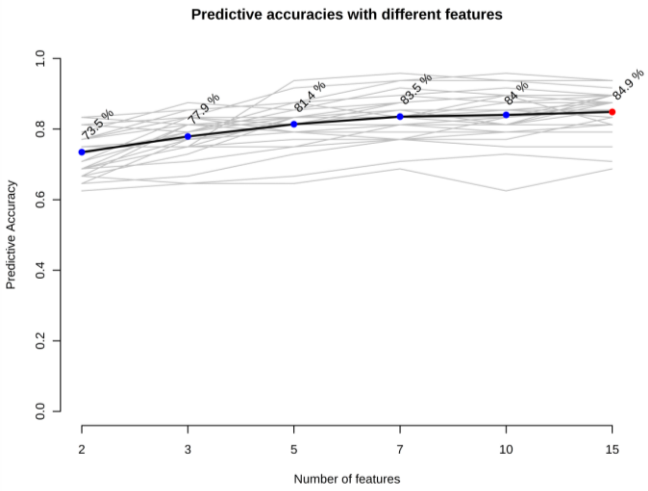

N vs dnA+MHA

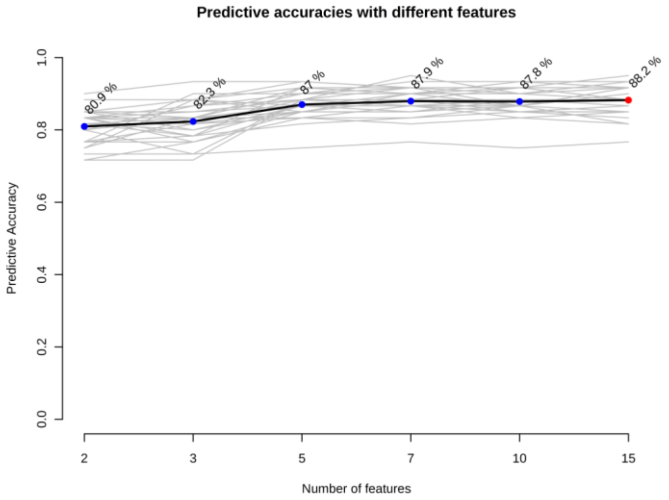

Supplementary Figure 2. Improvement of predictive accuracy when including all the molecules. All transitions per protein are included (supplementary table 8).

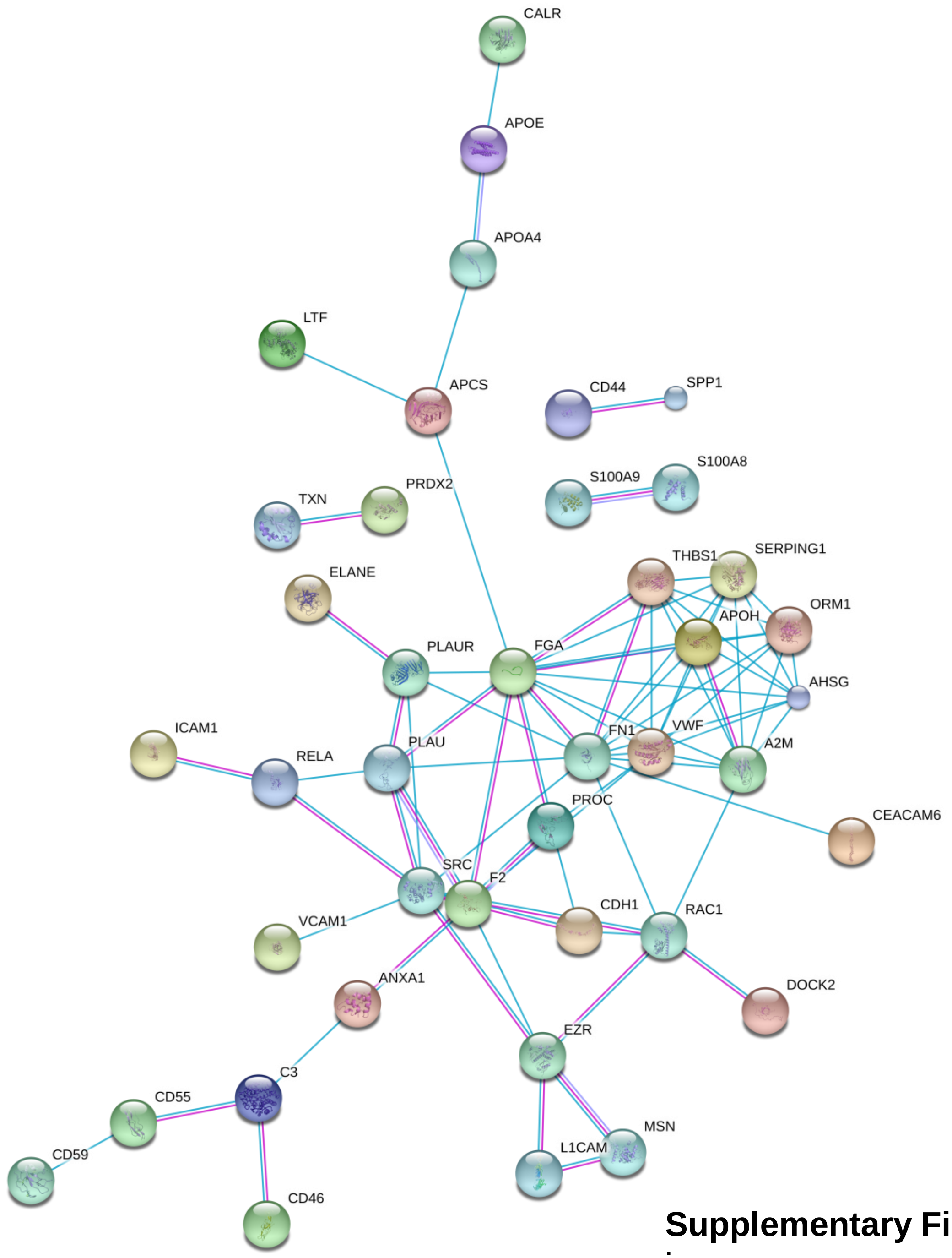

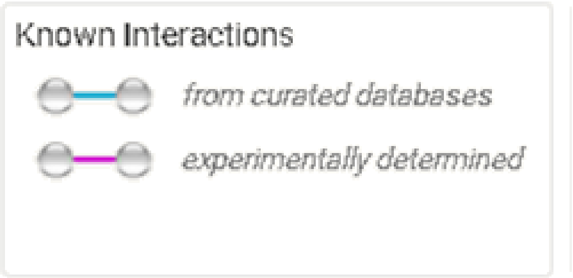

Supplementary Figure 3. Among the 70 proteins identified related to immune response, 41 proteins are interconnected as shown here.
